# Echocardiography-guided subxiphoidal transapical TAVR in a challenging anatomy: a case report

**DOI:** 10.1186/s13019-020-01181-4

**Published:** 2020-06-09

**Authors:** Andres Beiras-Fernandez, Angela Kornberger, Martin Geyer, Alexander Tamm, Ralph Stephan von Bardeleben

**Affiliations:** 1 Department of Cardiothoracic and Vascular Surgery, University Medical Center Mainz (Johannes Gutenberg-University Mainz), Langenbeckstr. 1, 55131 Mainz, Germany; 2 Center for Cardiology, Cardiology I, University Medical Center Mainz (Johannes Gutenberg-University Mainz), Mainz, Germany

**Keywords:** Transcatheter aortic valve replacement, Modified transapical access, Subxiphoidal approach, Case report

## Abstract

**Background:**

Transcatheter aortic valve replacement (TAVR) via an antegrade transapical access (TA-TAVR) is largely reserved to cases not amenable to transfemoral TAVR. Challenges resulting from unusual thoracic anatomies may require special considerations in terms of the surgical access.

**Case presentation:**

We present a case of TA-TAVR through a subxiphoidal approach in a patient who had undergone extensive thoracic surgery 8 years previously.

**Conclusion:**

Our case demonstrates that unusual anatomic features should not discourage from TA-TAVR but may require unusual approaches designed on a case-to-case basis following careful interdisciplinary preparation and planning including adequate pre-operative diagnostics and imaging.

## Background

Transcatheter aortic valve replacement (TAVR) has become the standard method for the treatment of aortic valve stenosis in intermediate and high risk patients and is mostly performed via a retrograde transfemoral route (TF-TAVR). The antegrade transapical access (TA-TAVR) is largely reserved – with some degree of local variability and preference - to cases not amenable to TF-TAVR because the iliofemoral arteries are too narrow, tortuous, diseased or calcified [1–3]. In cases scheduled for TA-TAVR, challenges in terms of the access may result from extreme obesity, unusual anatomic characteristics or changes caused by prior thoracic surgery. We present a modified subxiphoidal access that may be used in cases where the heart has shifted rightwards such as in our patient, whose anatomy was very distinct following previous thoracic surgery.

## Case presentation

We present the case of a 76 year old female who was referred for TAVR with an extensive medical history including right upper and middle lobectomy for bronchial carcinoma 8 years previously, long-standing GOLD IV COPD with steroid treatment and recurrent exacerbations, coronary three vessel disease with a history of several myocardial infarctions and repeated PCI, diabetes mellitus, carotid artery disease, and peripheral artery disease that had been treated by stenting and bypass grafting. Her Euroscore II was 3.92 / log Euroscore I 26.45%.

**Fig. 1:**
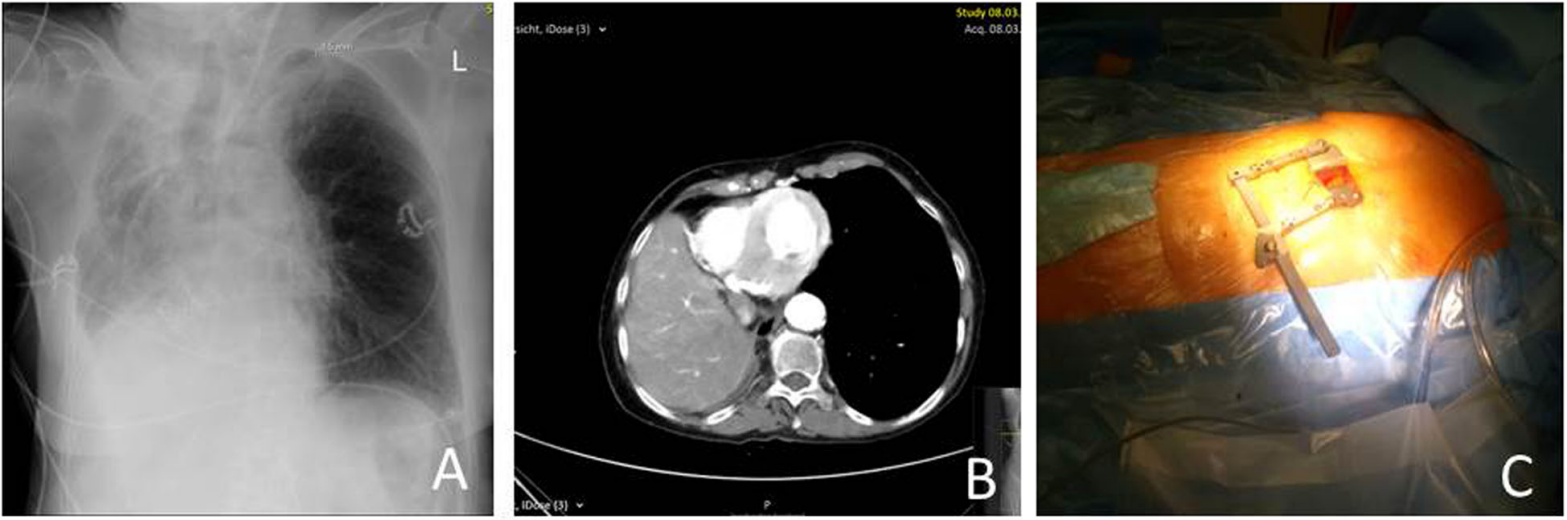
**a** and **b** showing rightward shift of the heart. The apex is located below the sternum. **c** shows the subxiphoidal incision through which TA-TAVR was performed. Arrow on the patient’s chest marks location of the apex as identified by the surgeon by means of transthoracic echocardiography

TF-TAVR was not feasible due to multiple stenoses of the iliofemoral vessels with presence of several bypasses and a stent protruding from the right common iliac artery into the iliac bifurcation. The subclavian, direct aortic and trans-caval approaches were considered but deemed not feasible due to a stenosis of the left subclavian artery, a heavily calcified, abnormally located ascending aorta, and a heavily calcified abdominal aorta with an acute-angled kink and chronically dissected aneurysm, respectively. CT additionally showed that the mediastinal structures were dislocated rightwards to such a degree that the apex could not be reached through the typical anterolateral incision used for TA-TAVR (Fig. 1a, b). The apex was therefore accessed through an untypical subxiphoidal incision without affecting the sternum or the ribs (Fig. 1c). Pre-incision transthoracic echocardiography allowed us to locate the apex directly below the xyphoid, allowing the incision to be kept extremely small. TA-TAVR using a 23 mm Sapiens 3 (Edwards Lifesciences, Irvine, USA) valve was performed in standard fashion without complications. After the procedure, the patient took a prolonged course due to pneumonia requiring re-intubation and was finally discharged after an ICU stay and total postoperative hospital stay of 6 and 17 days, respectively. Follow-up after 4 months showed her in a good functional condition with a mean aortic valve gradient of 7 mmHg and no relevant aortic regurgitation.

## Discussion and conclusions

In conclusion, unusual anatomic features should not discourage from TA-TAVR but may in certain cases be compensated by unusual approaches designed on a case-to-case basis. Pre-incision transthoracic echocardiography allows precise identification of the incision site so that the dimensions of the incision can be kept to a minimum. Alternatives to TA access these cases, including TAVR through carotid, subclavian or trans-caval access should be considered depending on patient anatomy and the type of bioprosthesis to be implanted. Treatment at a high-volume center and extensive implanter experience in TA-TAVR and other transapical procedures are mandatory for success.

## Data Availability

The datasets used and/or analyzed during the current study are available from the corresponding author on reasonable request.

## Abbreviations

TA: Transapical
TF: Transfemoral
TAVR: Transcatheter aortic valve replacement
